# Diagnostic Accuracy and Measurement Properties of Instruments Screening for Psychological Distress in Healthcare Workers—A Systematic Review

**DOI:** 10.3390/ijerph20126114

**Published:** 2023-06-13

**Authors:** Lima M. Emal, Sietske J. Tamminga, Sanja Kezic, Frederieke G. Schaafsma, Karen Nieuwenhuijsen, Henk F. van der Molen

**Affiliations:** Amsterdam UMC Location University of Amsterdam, Amsterdam Public Health, Public and Occupational Health, Meibergdreef 9, 1105 AZ Amsterdam, The Netherlands

**Keywords:** health personnel, self-reported stress, burnout, psychological, psychometrics, diagnostic criteria

## Abstract

Background: Instruments with sufficient diagnostic accuracy are better able to detect healthcare workers (HCWs) who are at risk of psychological distress. The objective of this review is to examine the diagnostic accuracy and measurement properties of psychological distress instruments in HCWs. Methods: We searched in Embase, Medline and PsycINFO from 2000 to February 2021. We included studies if they reported on the diagnostic accuracy of an instrument. To assess the methodological quality of the studies with regard to diagnostic accuracy, we used the Quality Assessment of Diagnostic Accuracy Studies and, for the measurement properties, the Consensus-based Standards for the Selection of Health Measurement Instruments (COSMIN). Results: Seventeen studies reporting on eight instruments were included. Overall, the methodological quality assessing the diagnostic accuracy and measurement properties was low, specifically for items addressing the domain ‘index test’. The items addressing ‘reference standard’, ‘time and flow’ and ‘patient selection’ were mostly unclear. The criterion validity of the single-item burnout, the Burnout–Thriving Index, and the Physician Well-Being Index (PWBI) was sufficient, with area under the curve ranging from 0.75 to 0.92 and sensitivity 71–84%, respectively. Conclusion: Our findings indicate that it is questionable whether screening for HCWs at risk of psychological distress can be performed sufficiently with the included instruments due to the low numbers of studies per instrument and the low methodological quality.

## 1. Introduction

In the working population, healthcare workers (HCWs) are more often affected by psychological distress than the general population [1]. Psychological distress encompasses case definitions with various concepts and constructs, ranging from stress symptoms to severe mental health disorders such as burnout, depression and anxiety. Psychological distress, in this review, is defined as a discomforting emotional state experienced by an individual in response to specific (work) demands, often resulting in disability for work [2]. Psychological distress in HCWs is often caused by a combination of factors such as effort–reward imbalance, high job demands, organizational injustice, lack of social support, high emotional demands and lack of decision-making authority, personality traits and personal circumstances [3]. The consequences of psychological distress for the health and well-being of HCWs are significant. That is, severe psychological distress can lead to a higher risk of coronary heart disease, burnout, depression and anxiety [4]. Psychological distress is also associated with adverse effects on the work functioning of HCWs. For instance, in the quality of patient consultations, these include an increase in sickness-related absence, medical errors and higher societal costs [5]. Due to the consequences of psychological distress, it is more difficult for healthcare organizations to recruit and retain a sufficient workforce, which possibly impacts the shortage of HCWs [6]. Therefore, in order to avert the adverse consequences for themselves, patients, the healthcare sector and society at large the prevention in psychological distress in HCWs is important.

A strategy to prevent psychological distress includes screening HCWs for psychological distress. The primary goal of screening is to detect signs, symptoms and risk factors of a disease at an early stage in large numbers of individuals. The main goal is to prevent individuals contracting the actual disease and to improve their health by offering treatment and/or support [7]. Screening for psychological distress in HCWs provides the possibility of directing HCWs to personalized preventive interventions, with the intent to increase their awareness and motivate them to take action [8]. Screening for psychological distress is preferably conducted with validated instruments that make early detection of psychological distress more effective [9].

In the last decade, the number of intervention studies aimed at the prevention of or reduction in psychological distress in HCWs has increased substantially. In many of these interventions, instruments have been used to screen for HCWs at risk of psychological distress and offer them preventive measures [5,10]. However, it is not clear how accurate these instruments used in the interventions are in screening for psychological distress in HCWs. There is a distinction between diagnosis and screening. That is, a diagnosis is aimed at identifying a specific condition in an individual who has symptoms, while screening is aimed at identifying a potential disease in individuals without symptoms [7]. Despite this distinction, an important aspect of both a screening instrument as well as a diagnostic instrument is diagnostic accuracy. However, this distinction between diagnosis and screening is not always clear; there is some overlap between the two, as some instruments can serve as both a screening as well as a diagnostic instrument, depending on the context in which they are used [11,12]. For example, screening can serve as a first step in the diagnostic process; in regard to psychological distress, this is relevant. For example, in Dutch occupational health checks, the four-dimensional symptom questionnaire (4DSQ) is commonly used to screen psychological distress in the working population. This screening instrument helps the occupational physician with diagnostic information [13].Diagnostic accuracy, sensitivity and specificity are important measures of the performance of an instrument.

The diagnostic accuracy of an instrument also largely depends on the quality of the study [14]. The screening ability of instruments is related to the measurement property of the ‘criterion validity’, and the most commonly used measures are sensitivity and specificity. Sensitivity is the degree to which an instrument minimizes false negatives and specificity is the degree to which an instrument minimizes false positives [12]. In addition to the criterion validity, the measurement properties in terms of the properties of reliability, validity and interpretability are also important in determining the adequacy of the instrument [13].

To the best of our knowledge, no systematic reviews have been conducted that investigated the diagnostic accuracy and measurement properties of instruments screening for psychological distress in HCWs. Therefore, to determine which instrument can adequately screen for psychological distress among HCWs, it is important to investigate the diagnostic accuracy of the instruments as well as the measurement properties. The objective of this review is to examine the diagnostic accuracy and measurement properties of psychological distress instruments in HCWs.

## 2. Methods

The review was performed according to the checklist of preferred reporting items for systematic reviews and meta-analyses (PRISMA). To assess the risk of bias of the evaluation of diagnostic accuracy of the instruments, we used the QUADAS-2 tool (University of Bristol, Bristol, UK) [12]. To assess the measurement properties of the instruments, we used the Consensus-based Standards for the Selection of Health Measurement Instruments (COSMIN) (COSMIN.nl (accessed on 28 July 2022)) [13]. This review utilizes definitions according to the consensus-based terminology of the COSMIN which means that we assessed measurement properties in the included studies according to the COSMIN taxonomy and not according to how authors themselves defined it [13].

### 2.1. Information Sources and Search Strategy

Studies were retrieved by searching in Medline, Embase and PsycINFO in February 2021. The search was conducted by a clinical librarian. The literature search was based on combined searches for concepts and their similar and related terms (see Supplementary File S1 Search strategy).

### 2.2. Eligibility Criteria

Studies published between 2000 and 2020 in peer-reviewed journals in English or Dutch were included if: (1) they reported on a specific instrument aimed at early detection of psychological distress in HCWs or a generic instrument used for the early detection of psychological distress which was validated in HCWs, and (2) the aim was to study the screening ability of the instrument for psychological distress in HCWs. Studies were excluded when: (1) they utilized an objective measure of stress such as cortisol, (2) the study population included informal caregivers or students, and (3) they examined the psychometric properties of an instrument that measured risk factors of psychological distress instead of stress symptoms.

If an included study reported on the examination of the screening ability of an instrument, we included all other studies that also studied other measurement properties of that specific instrument to provide a full overview of measurement properties of that included instrument.

### 2.3. Selection Process

All of the retrieved studies were independently screened for eligibility on the basis of title and abstract by at least two authors against the inclusion and exclusion criteria specified above. For screening titles and abstracts we used the web-based screening tool Rayyan. One author (L.M.E.) screened all studies. Next, half of the studies were randomly assigned for screening to one author (S.J.T.) and the other half to other authors (S.K., F.G.S., K.N., H.F.v.d.M.). Conflicts between the authors about inclusion on the basis of title and abstract amounted to 3%. Thereafter, the selected studies were screened independently by two authors (L.M.E., S.J.T.) based on the full texts. Disagreements were resolved by discussion amongst the authors (L.M.E., S.J.T.). We used a two-step process to include eligible studies for this review. We first included studies on the basis of the selection criteria, and then we searched for additional studies that examined other measurement properties of the instruments included.

### 2.4. Data Collection Process

For the assessment of the methodological quality of the study and the rating of measurement properties, we extracted the following data if applicable and reported in the study: criterion validity, internal consistency, reliability, hypotheses testing and structural validity. The author L.M.E. independently performed data extraction for all studies, which was checked by S.J.T. Disagreements between the two authors were discussed to achieve the final decisions.

### 2.5. Assessment of the Methodological Quality of the Studies

To assess the methodological quality of the studies examining diagnostic accuracy of the instrument, we used the QUADAS-2 [12]. We assessed only the diagnostic accuracy of studies that examined the criterion validity. QUADAS-2 covers four domains: (1) patient (worker) selection, (2) index test, (3) reference standard and (4) flow and timing. Each domain includes signaling questions to assess the risk of bias (Supplementary File S2). The signaling questions are related to whether the patient (worker) selection could have introduced bias, to whether the conduct or interpretation of the index test and reference standard could have introduced bias, or to whether the patient flow could have introduced bias. Secondly, the applicability of the (1) patient (worker) selection, (2) index test and (3) reference standard was judged. Eventually, the domains and the concerns of applicability were rated as ‘high risk’, ‘low risk’ or ‘unclear’. The four domains and applicability taken together provide the risk of bias of the diagnostic accuracy [12].

The COSMIN Risk of Bias checklist was used to assess the methodological quality of the studies on measurement properties [13]. The COSMIN Risk of Bias checklist is a tool developed to assess the methodological quality of single studies on measurement properties of instruments. This tool contains standards referring to design requirements and preferred statistical methods of studies on measurement properties. For each measurement property, a COSMIN box was developed, containing all standards needed to assess the quality of a study on that specific measurement property. The methodological quality of the study is rated ‘very good’, ‘adequate’, ‘doubtful’ or ‘inadequate’. The overall score for each box is determined by the lowest item score, meaning that if the lowest score for the box is rated as ‘inadequate’ the whole box is rated as such.

### 2.6. Rating of the Measurement Properties

We rated the results of the measurement properties per instrument across studies, with the COSMIN criteria for good measurement properties [15]. For example, for criterion validity, if convincing arguments are presented that the standard used really is ‘gold’, and if the correlation with the gold standard is at least 0.70, it is rated as sufficient. We rated the properties for each study separately, as they report on different study populations and results differ. The results for the measurement properties were rated as sufficient (+), insufficient (−), or indeterminate (?). Since the COSMIN criteria for good measurement properties have no criteria for sufficient sensitivity, we rated the results as follows: a value of >85% was considered high, 70–85% was considered moderate, and <70% was considered low [14].

### 2.7. Synthesis Methods

Since our primary aim was to review the diagnostic accuracy of psychological distress screening instruments, we decided a priori that we would perform a meta-analysis on diagnostic accuracy only when at least two studies assessed the diagnostic accuracy of the same measurement instrument. Since this was not the case, we have not conducted a meta-analysis.

## 3. Results

### 3.1. Study Selection

**Figure 1:**
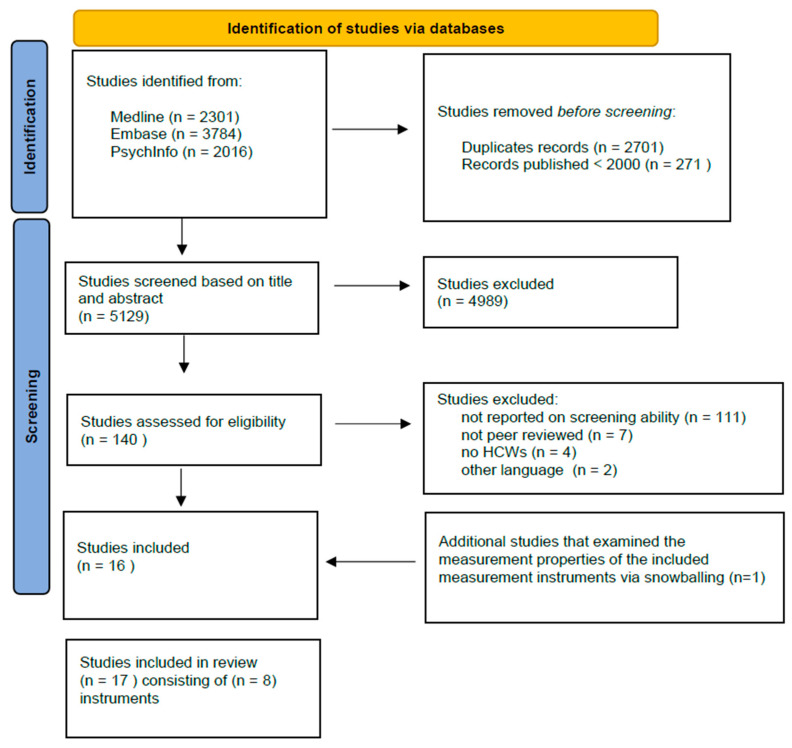
Study selection flowchart.

After removal of duplications and deletion of studies that were published before 2000, 5129 studies remained, of which the titles and abstracts were screened (see Figure 1). Eventually, 140 studies were assessed for eligibility at the level of full-text screening. After applying the inclusion and exclusion criteria, 17 studies were retained containing 8 measurement instruments.

### 3.2. Study Characteristics

Seventeen studies covering eight instruments screening for psychological distress in HCWs were included in this review [16,17,18,19,20,21,22,23,24,25,26,27,28,29,30,31,32] (Table 1 and Supplementary File S3). These are the work functioning screener-healthcare (WFS-H) [17], the Burnout Battery [18], the Physician well-being index (PWBI) [20,21,22,23] the Professional quality of Life (ProQOL) [16,25,26,27,28,30], the Burnout–thriving index [26], the single-item burnout [19,27,29,32] and the Professional fulfilment index (PFI) [24,31]. The sample sizes of the HCWs included in the studies varied from 249 to 7288. The most common occupations addressed were nurses and physicians. The mean age of the participating HCWs varied between 41 and 55 years, and mostly women participated.

### 3.3. Assessment of the Methodological Quality of the Studies

The assessment of the quality of studies examining the diagnostic accuracy of the instruments is described in Table 2. None of the instruments had an overall ‘low risk of bias’ judgement. The patient (worker) selection of the Burnout Battery and PWBI were judged as ‘low risk’ and the single-item of burnout (BO) Rohland as ‘potential high risk’. The patient selections of the seven instruments were judged as ‘unclear’. The index test for Hansen’s single-item burnout was judged as ‘low risk’ [27] and remaining instruments as ‘high risk’. The flow and timing of the WFS-H [17], Burnout inventory index [26] and PFI [31] were judged as ‘low risk’, and other five instruments as ‘unclear’.

All studies were judged as ‘high concern regarding applicability’ for the reference standard, except for Hansen’s single-item burnout [27], because all instruments were examined in a subgroup of HCWs such as physicians or nurses. The concerns regarding applicability for the patient selections and index tests of all instruments were judged as ‘low’ because the populations of those instruments matched our review question. Another reason for low concern was that there was no variation between the reference standard and index tests in these studies, both of them being questionnaires.

The assessment of the methodological quality of the studies on measurement properties is described in Table 3 and Table 4. The two most frequently assessed measurement properties were criterion validity and internal consistency. The methodological quality of studies assessing criterion validity was rated as ‘doubtful’, the main reason being that it is unclear whether the criterion used can be considered an adequate gold standard [16,17,18,20,21,22,23,26,28,29,30,31,32].

Four studies assessed the hypotheses testing of WFS-H [17], PFI [24,31] and ProQOL [25,28,30]. The methodological quality of studies examining the PFI and ProQOL [25,28,30] was rated as ‘inadequate’, due to flaws in the study design. These two studies failed to formulate hypotheses including the expected direction or a clear description of the constructs measured by the comparator instrument. The methodological quality of the study of Boezeman [17] was ‘very good’; authors formulated hypotheses, the comparator instruments were mentioned and the results were in accordance with the hypotheses (Table 3).

Six studies assessed the internal consistency of the ProQOL and PFI (Table 4). The quality of these studies was rated as ‘very good’ [24,25,28,30,31]. The studies used an adequate study design and used preferred statistical methods. Each scale or subscale is unidimensional, the sample consisted of more than 100 respondents, the manner in which missing items were handled was clearly described and all studies calculated the Cronbach’s alpha. Trockel et al. [31] also examined the reliability of the PFI; however, we rated the methodological quality of this study as ‘inadequate’ because the authors failed to conduct two measurements. Seven studies examined construct validity; the quality of the studies ranged from ‘very good’ to ‘inadequate’. The construct validity of the study of Hemsworth et al. [28] and Samson et al. [30] investigating the ProQOL and the PFI of Fadare [24] was ‘very good’. However, the quality of the study of Trockel [31] and the sub-sample of the Brazilian sample of Galiana [25] was rated as ‘inadequate’.

### 3.4. Rating of the Measurement Properties

The criterion validity of the single-item burnout [18], the Burnout–thriving index [26], the PWBI and 9 item PWBI were rated as ‘sufficient’, with an area under the curve >0.70, correlations >0.70 and sensitivity >80% (Table 3). In the studies of Hansen and Rohland [29] the results for criterion validity were ‘insufficient’, because these instruments correlated poorly with the reference standard. The criterion validity of the remaining seven studies was inconsistent, as the area under the curve for the sub-constructs quality of life and burnout of PWBI were sufficient >0.80, but fatigue and suicidal ideation were insufficient 0.62–0.65. The results of the hypotheses testing of the WFS-H were considered sufficient (Table 3 and Table 4).

The internal consistency for the instrument PFI was ‘sufficient’, with Cronbach’s alphas > 0.70 [24,31]. The internal consistency of the ProQOL was ‘sufficient’ and consistent throughout the studies. All studies reported Cronbach’s alpha higher than 0.70, except for the sub-construct burnout. The results for the structural validity of the ProQOL were inconsistent. The comparative fit index was ‘sufficient’ > 0.95; however, it was ‘insufficient’ in other studies [24,28]. The structural validity of the PFI was also inconsistent [24,31] (Table 3 and Table 4).

## 4. Discussion

### 4.1. Main Findings and Interpretation of Results

In this systematic review, we found seventeen studies that examined eight different instruments. Our results demonstrate that, due to the low number of included studies per instrument and the poor quality of the included studies, we are uncertain whether the diagnostic accuracy of instruments screening for psychological distress is sufficient. This lack of information makes it difficult to select the ‘best’ instrument for screening HCWs at risk of psychological distress. However, the few available measurement properties across studies were mostly sufficient, such as the area under the curve of the PWBI [21], the sensitivity of the Burnout–thriving index [26] and the correlation of the single-item burnout [29].

The evaluation of the diagnostic accuracy of an instrument depends on the measurement properties of the instrument itself and on the quality of the study. Studies that do not meet methodological standards usually over- or under-estimate the indicators of diagnostic accuracy and they limit the applicability of the results of the study [33]. For example, in our review, we found that in twelve of the thirteen studies, the sensitivity and specificity were not specified beforehand. A lack of specification beforehand may lead to a cut-off point being chosen with high sensitivity and specificity. This data-driven method is often used to maximize diagnostic accuracy, but studies doing so generally overestimate the sensitivity and specificity [34].

The methodological quality of the studies examining diagnostic accuracy also depend on the gold standard. The gold standard is the best available method for identifying people that have the target condition [35]. However, no gold standard exists for screening for psychological distress in HCWs. In this review, all the studies included used gold standards with doubtful diagnostic accuracy themselves. This finding indicates that it is difficult to assess whether preventive interventions that aim to screen HCWs at risk are doing so adequately. This likely influences the effectiveness of reaching HCWs at risk for psychological distress and offering them supportive interventions in a timely manner.

### 4.2. Comparison to Other Literature

To the best of our knowledge, the diagnostic accuracy of instruments screening for psychological distress in HCWs has not yet been investigated in systematic reviews. However, the diagnostic accuracy of screening instruments in other populations and other psychological concepts such as depression and anxiety has been studied [36]. One systematic review found evidence of moderate quality for three depression screening instruments with sufficient diagnostic accuracy to screen for depressive symptoms in the general population [36]. The main reason is probably that for depression, there is much greater consensus about the gold standard for diagnosing depression. The gold standard in this case is deemed to be a clinical interview or the Diagnostic and Statistical Manual of Mental Disorders (DSM-5); this increases the methodological quality of the studies [37]. Furthermore, this review had more studies per instrument, which made it possible to conduct a meta-analysis.

Even though at this moment we cannot recommend an instrument for screening psychological distress that has been validated specifically in HCWs, other screening instruments can adequately screen for psychological distress in other populations. One study that investigated the distress scale of the 4DSQ concluded that this instrument could be used to identify employees at risk of psychological distress [11]. The Need for Recovery Scale is also reported as an adequate instrument to screen for psychological distress in the general working population [34]. 

### 4.3. Strengths and Limitations

Our systematic review adds to the literature by providing a structured and complete overview of the diagnostic accuracy of instruments screening for psychological distress in HCWs and the methodological quality of the studies assessing them. Additionally, we designed a highly sensitive search strategy with the help of a clinical librarian.

This study does have a number of limitations. The QUADAS-2 was developed to examine the diagnostic accuracy of instruments; however, it is also applicable to evaluate the screening ability of instruments. The QUADAS-2 takes important aspects into account regarding the study design, such as selection bias, reference standard accuracy, and patient flow, which are also relevant for the screening ability of instruments. The QUADAS-2 was developed to assess the diagnostic accuracy of objective measurement instruments (e.g., CT scan) which makes this tool for self-reported instruments less suitable. For example, when we assessed the applicability of the index test, all studies were judged as ‘low risk of bias’ since the order of taking the index and reference test is not important for psychological distress.

Finally, we searched for studies published after 2000, but some studies that examined the screening ability were published well before 2000. On the other hand, this probably did not have a great impact on our results because we applied snowballing of references of included studies.

### 4.4. Implications of Results for Practice, Policy and Future Research

Without high-quality evidence that demonstrates sufficient diagnostic accuracy, we cannot recommend an instrument that sufficiently screens for psychological distress in HCWs. In this review, the domain index test for twelve of the thirteen studies was rated as ‘high risk of bias’. For example, the threshold of the sensitivity and specificity was not pre-specified which can lead to over-estimation of the diagnostic accuracy. We currently recommend that researchers or clinicians should consider using psychological distress screening instruments that have been validated in other populations, with sufficient diagnostic accuracy, such as the distress scale of the four-dimensional symptom questionnaire or the Need for Recovery Scale. Future research should focus on the improvement of the methodological quality of studies examining diagnostic accuracy.

To overcome problems in systematic flaws in the study designs, we recommend that researchers use the Standards for Reporting of Diagnostic Accuracy Studies (STARD). The STARD provides clear guidance for planning reports of diagnostic accuracy studies. Furthermore, we also recommend researchers use the QUADAS-2 tool; this tool is complimentary to the STARD and, in this manner, diagnostic accuracy studies with design deficiencies can reduce or overcome biased results [38]. For example, the order of testing the index test and reference standard is important because knowledge of the reference standard may be influenced by the interpretation of the index test results.

Finally, in this review, studies mostly measured and reported on the area under the curve (AUC); however, this tells us nothing about the sensitivity and specificity. A test may have a much higher sensitivity than another test, even though they both have the same AUC [33]. Therefore, researchers that examine the diagnostic accuracy of an instrument should always examine the sensitivity and specificity of screening instruments. Thus, this indicates that beside the low methodological quality of the studies, we also do not have complete information about the diagnostic accuracy measures (e.g., sensitivity/specificity) of the instruments.

## 5. Conclusions

Our systematic review revealed that it is questionable whether screening HCWs at risk for psychological distress can be performed adequately with the included instruments due to the low methodological quality of the included studies and the low number of studies per instrument. These results may negatively impact the effectiveness of interventions screening HCWs at risk of psychological distress.

## Figures and Tables

**Table 1:** Characteristics of the study population, instrument administration, reference standard and measurement properties.

			Study Population				Instrument Administration				Reference Standard	Measurement Properties Examined
Instrument	Author/Year	Construct	Sample Size	Occupation	Mean Age (SD)	Female n (%)	Mode of Administration	Language	Setting	Response Rate		
WFS-H	Boezeman et al., 2016 [17]	Unimpaired work functioning	249	Nurses	47 (12)	209 (84%)	Self-administration	Dutch	Academic Medical Center	24%	NWFQ, EWPS, 4DSQ	Criterion validity
Burnout battery	Deng et al., 2017 [18]	Burnout syndrome	538	Physicians, nurses	nr	430 (80%)	Self-administration	nr	University, cancer and municipal hospitals	81%	MBI-HSS, Doctors’ Job Burnout Questionnaire.	Criterion validity
Physicians well-being Index	Dyrbye et al., 2013 [22]	Multi-dimensions of distress	7288	Physicians	nr	1530 (21%)	Self-administration	English	nr	26%	MBI-personal accomplishment	Criterion validity
	Dyrbye et al., 2014 [23]	Multi-dimensions of distress	1701	Residents	nr	918 (54%)	Self-administration	English	nr	23%	Mental QOL and level of fatigue on a standardized linear analogue scale.	Criterion validity
	Dyrbye et al., 2019 [20]	Multi-dimensions of distress	976	Nurses	52 (11)	892 (92%)	Self-administration	English	nr	47%	Overall QOL on a standardized linear analogue scale. MBI-depersonalization MBI- emotional exhaustion	Criterion validity
			60	Physician assistants	46 (11)	409 (69%)				30%		
9-item well-being index	Dyrbye et al., 2016 [21]	Multi-dimensions of distress	5392	General working population, physicians	nr	2458 (46%)	Self-administration	English	nr	nr	Overall QOL on a standardized linear analogue scale. MBI-depersonalization MBI-emotional exhaustion	Criterion validity
Professional quality of Life Scale	Ang et al., 2020 [16]	Compassion satisfaction, compassion fatigue	1338	Nurses	nr	1250 (93%)	Self-administration	nr	Academic medical hospital	28%	PANAS	Internal consistency, reliability, structural validity, hypotheses testing
	Galiana et al., 2017 [25]	Compassion satisfaction, compassion fatigue	385	Physicians, nurses, Psychologists, nursing assistants, social workers, other	nr	300 (78%)	Self-administration	Spanish	nr	nr	na	Internal consistency, structural validity.
		Compassion satisfaction, compassion fatigue	161	Physicians, nurses, psychologists, nursing assistants, social workers, other	nr	143 (89%)	Self-administration	Portuguese	nr	nr	na	Internal consistency, structural validity.
	Hemsworth et al., 2018 [28]	Compassion satisfaction, compassion fatigue	273	Australian nurses	40 (nr)	253 (93%)	Self-administration	English	Hospital	21%	na	Internal consistency, structural validity, hypothesis testing.
			303	Canadian nurses	40 (nr)	263 (87%)				30%		
			651	Canadian palliative care workers	53 (nr)	553 (85%)				67%		
	Samson et al., 2016 [30]	Compassion satisfaction, compassion fatigue	377	Physicians	48 (10)	20%	Self-administration	Hebrew	Primary health and palliative care setting	35%	Shirom-Melamed burnout measurement	Internal consistency, structural validity, hypothesis testing, criterion validity
				Nurses		80%						
				Social workers		nr						
Burnout-thriving index	Gates et al., 2019 [26]	Burnout	1365	Physicians, fellows, residents, medical students, nurses	41 (nr)	979 (72%)	Self-administration	English	Hospital	45%	MBI	Criterion validity
Single item burnout	Dolan et al., 2015 [19]	Burnout	5404	Physicians, nurse practitioners, physician assistants, clinical associates administrative clerks.	nr (nr)	nr (nr)	Self-administration	English	Primary care clinic	25%	MBI- emotional exhaustion	Criterion validity
	Hansen et al., 2010 [27]	Burnout	740	Nurse oncologist, palliative care physician, other health professionals, research and administration	46 (10)	499 (78%)	Self-administration	English	Various medical institutions	56%	MBI-HSS emotional exhaustion, single-item self-defined burnout scale	Criterion validity
	Rohland et al., 2004 [29]	Burnout	307	Physicians	44 (nr)	78 (26%)	Self-administration	English	Alumni of the Texas Tech University school of medicine	43%	MBI	Criterion validity
	Waddimba et al., 2016 [32]	Burnout	308	Rural physician/non-physician practitioners	49 (nr)	141 (46%)	Self-administration	English	Academic medical centre, community hospitals, clinics and school-based health centres	65%	MBI-HSS emotional exhaustion, depersonalization, The Single-item Burnout, measure from the Physician Work Life Study	Criterion validity
Professional fulfillment index	Trockel et al., 2018 [31]	Professional wellbeing	250	Physicians	nr	123 (49%)	Self-administration	English	Academic medical centre	nr	MBI-HSS the one-item self-defined burnout measure, sleep-Related Impairment Depression, and Anxiety scales, WHOQOL-BREF	Internal consistency, reliability, structural validity, hypothesis testing, criterion validity.
	Fadare et al., 2021 [24]	Professional wellbeing	4716	Pharmacists	44(13)	3059(65%)	Self-administration	English	nr	56%	nr	Internal consistency, Structural validity

Abbreviations: na, not applicable; nr, not reported; WFS-H, work functioning screener; QOL, quality of life; NWFQ, Nurse work functioning questionnaire; EWPS, 25-item Endicott Work Productivity Scale; 4DSQ, four-dimensional symptoms questionnaire; MBI-HSS, Maslach Burnout Inventory-Human Services Survey; PANAS, Positive and Negative Affect Schedule; WHOQOL-BREF, WHO quality of life.

**Table 2:** The risk of bias of the evaluation of diagnostic accuracy of the measurement instruments that examined the criterion validity.

Instrument/Author/Year	Domains				Applicability Concerns		
	Patient (Worker) Selection	Index Test	Reference Standard	Flow and Timing	Patient Selection	Index Test	Reference Standard
WFS-H/Boezeman et al., 2016 [17]	Unclear	High risk	Unclear	Low risk	Low risk	Low risk	High risk
Burnout Battery/Deng et al., 2017 [18]	Low risk	High risk	Unclear	Low risk	Low risk	Low risk	High risk
Physician well-being index/Dyrbye et al., 2013 [22]	Unclear	High risk	Unclear	Unclear	Low risk	Low risk	High risk
Physician well-being index/Dyrbye et al., 2014 [23]	Low risk	High risk	Unclear	Unclear	Low risk	Low risk	High risk
9 item physician well-being index/Dyrbye et al., 2016 [21]	Unclear	High risk	Unclear	Unclear	Low risk	Low risk	High risk
Physician well-being index/Dyrbye et al., 2019 [20]	Unclear	High risk	Unclear	Unclear	Low risk	Low risk	High risk
Professional quality of life/Samson et al., 2016 [30]	Unclear	High risk	Unclear	Unclear	Low risk	Low risk	High risk
Burnout-thriving index/Gates et al., 2011 [26]	Unclear	High risk	Unclear	Unclear	Low risk	Low risk	Low risk
Single Item of BO/Dolan et al., 2015 [19]	Unclear	High risk	Unclear	Unclear	Low risk	Low risk	High risk
Single Item of BO/Hansen et al., 2010 [27]	Unclear	Low risk	Unclear	Unclear	Low risk	Low risk	Low risk
Single Item of BO/Rohland et al., 2004 [29]	High risk	High risk	Unclear	Unclear	Low risk	Low risk	High risk
Single Item of BO/Waddimba et al., 2016 [32]	Unclear	High risk	Unclear	Unclear	Low risk	Low risk	High risk
PFI/Trockel et al., 2018 [31]	Unclear	High risk	Unclear	Low risk	Low risk	Low risk	High risk

**Table 3:** Methodological quality of the studies and rating the results of the measurement properties hypotheses testing and criterion validity.

			Hypotheses Testing		Criterion Validity				
Instrument (Ref)	Sub-Samples	Subscales	Methodological Quality of the Studies	Results Measurement Property (Rating) A. Hypotheses Confirmed 75%	Methodological Quality of the Studies	Results Measurement Property (Rating) A. Area under the Curve B. Pearson Correlations C. Spearman Correlations D. Sensitivity	Cut-Off-Point	Correlation with Scale	Comparator Measurement Instrument
WHF-S Boezeman et al., 2016 [17]	na	Cognitive aspects of task execution and general incident	Very good	A. WFS-H correlates moderately with productivity general health and distress (+)	Unclear	A. 0.79 (+) D. 71% (+)	0.25	na	na
		Avoidance behavior				A. 0.71 (+) D. 56% (−)	0.13		
		Conflicts and irritations with colleagues				A. 0.71 (+) D. 56% (−)	0.29		
		Impaired contact with patients and family				A. 0.64 (−) D. 42% (−)	0.19		
		Lack of energy and motivation				A. 0.82 (+) D. 76% (+)	0.32		
Burnout Battery Deng et al., 2017 [18]	na	na	na	na	Unclear	A. 0.76 (+) D. 68% (−)	3 bars	na	na
						A. 0.71 (+) D. 67% (−)	3 bars		
						A. 0.62 (−) D.55% (−)	3 bars		
Physician well-being index Dyrbye et al., 2013 [22]	na	Low quality of life	na	na	Unclear	A. 0.85 (+)	≥1/2 standard deviation	na	Standardized linear analogue scale MBI-PA
		High fatigue				A. 0.78 (+)	≥1/2 standard deviation		
		Suicidal ideation				A. 0.80 (+)	≥1/2 standard deviation		
Physician well-being index Dyrbye et al., 2014 [23]	na	Low mental quality	na	na	Unclear	D. 70% (+)	≥5	na	Standardized linear analogue scale
		High fatigue				D. nr	nr		nr
		Suicidal ideation				D. nr	nr		nr
9 item well-being indexDyrbye et al., 2016 [21]	Physician sample	High quality of life	na	na	Unclear	A. 0.80 (+)	≥1/2 standard deviation	na	Standardized linear analogue scale
		Low quality of life				A. 0.84 (+)	≥1/2 standard deviation		Standardized linear analogue scale
		Fatigue				A. 0.74 (+)	≥1/2 standard deviation		10-point linear analogue scale
		Burnout				A. 0.85 (+)	nr		MBI
Physician well-being Index Dyrbye et al., 2019 [20]	na	Quality of life	na	na	Unclear	A. 0.81 (+)	≥0.5 standard deviation	na	Linear analogue scale
		Fatigue				A. 0.62 (−)	≥0.5 standard deviation		Linear analogue scale
		Suicidal ideation				A. 0.65 (−)	Reported suicidal ideation within the past 12 months		Single item suicidal ideation
		Burnout				A. 0.77 (+)	EE ≥ 0.27 and DP ≥ 0.28		MBI
Professional quality of life scale Ang et al., 2020 [16]	na	na	Inadequate	?	na	na	na	na	na
Professional quality of life scale Hemsworth et al., 2018 [28]	Australian	Compassion satisfaction	Inadequate	?	na	na	na	na	na
		Burnout							
		Secondary traumatic stress							
	Canadian	Compassion satisfaction	Inadequate	?					
		Burnout							
		Secondary traumatic stress							
Professional quality of life scale Samson et al., 2016 [30]	na	Compassion satisfaction	Inadequate	?	Unclear	B. 0.72 (+)	na	na	UWES-9
		Burnout				B. 0.57 (−)			SMBM
		Secondary traumatic stress				B. 0.40 (−)			PDEQ
Professional fulfilment Trockel et al., 2018 [31]	na	Professional fulfilment	Inadequate	?	Unclear	A. 0.81 (+) D. 73% (+)	3.0	na	na
		Burnout				A. 0.85 (+) D. 72% (+)	3.0		
Single Item burnout Waddimba et al., 2016 [32]	na	na	na	na	Unclear	C. 0.72 (+)	na	Physicians work life with EE	MBI
						C. 0.41 (−)		Physicians work life with DP	
						C. 0.89 (+)		I feel burned out from work with EE	
						C. 0.81 (+)		I have become more callous with DP	
Single Item burnout Hansen et al., 2010 [27]	na	na	na	na	Unclear	B. 0.68 (−)	na	EE and self-defined burnout	MBI
Single Item burnout Rohland et al., 2004 [29]	na	na	na	na	Unclear	B. 0.64 (−)	nr	EE	MBI
						B. −0.26 (−)		PA	
						B. 0.32 (−)		DP	
Burnout thriving- Index Gates et al., 2019 [26]	na	na	na	na	Unclear	D. 84% (+)	nr	EE	MBI
						D. 81% (+)		PA	
						D. 81% (+)		DP	
Single item burnout Dolan et al., 2015 [19]	na	na	na	na	Unclear	A. 0.92 (+) B. 0.78 (+) D. 83% (+)	na	EE	MBI

Abbreviations: na, not applicable; nr, not reported; EE, emotional exhaustion; DP, depersonalization; PA, personal accomplishment; MBI, Maslach burnout inventory; UWES-9, Utrecht Work Engagement Scale; SMBM, Shirom-Melamed Burnout Measure; PDEQ, Peritraumatic Dissociative Experiences Questionnaire. Rating measurement properties: (+) sufficient, (−) insufficient, or (?) indeterminate. For sensitivity: >85% (++), 70–85% (+), and <70% was (−).

**Table 4:** Methodological quality of the studies and rating the results of the measurement properties internal consistency, reliability and structural validity per measurement instrument.

			Internal Consistency		Reliability		Structural Validity	
Instrument (Author/Year)	Sub-Samples	Subscales	Methodological of the Studies Quality	Results Measurement Property (Rating) Cronbach’s α (Rating)	Methodological Quality	Results (Rating)	Methodological Quality	Results (Rating) A. Comparative Fit Index B. Root Mean Square Error C. Standardized Root Mean Residuals
Professional quality of life Scale Galiana et al., 2017 [25]	Spanish sample	Compassion satisfaction	Very good	0.77 (+)	na	na	Very good	A. 0.94 (−) B. 0.07 (−)
		Secondary traumatic stress		0.78 (+)				
		Burnout		0.54 (−)				
	Brazilian sample	Compassion satisfaction	Very good	0.86 (+)	na	na	Inadequate	A. 0.94 (−) B. 0.08 (−)
		Secondary traumatic ttress		0.77 (+)				
		Burnout		0.65 (−)				
Professional quality of life ScaleAng et al., 2020 [16]	na	Compassion satisfaction	Very good	0.92 (+)	na	na	na	na
		Secondary traumatic stress		0.80 (+)				
		Burnout		0.78 (+)				
Professional quality of life ScaleHemsworth et al., 2018 [28]	Australian nurses	Compassion satisfaction	Very good	0.90 (+)	na	na	Very good	A. 0.99 (+) B. 0.73 (−)
		Secondary traumatic stress		0.82 (+)				A. 0.98 (+) B. 0.71 (−)
		Burnout		0.80 (+)				A. 0.82 (−) B. 0.15 (−)
	Canadian nurses	Compassion satisfaction	Very good	0.91 (+)	na	na	Very good	A. 0.99 (+) B. 0.71 (−)
		Secondary traumatic stress		0.85 (+)				A. 0.96 (+) B. 0.09 (−)
		Burnout		0.75 (+)				A. 0.82 (−) B. 0.15 (+)
	Canadian palliative nurses	Compassion satisfaction	Very good	0.87 (+)	na	na	Very good	A. 1.00 (+) B. 0.04 (−)
		Secondary traumatic stress		0.82 (+)				A. 0.98 (+) B. 0.07 (−)
		Burnout		0. 69 (−)				A. 0.85 (−) B. 0.17 (+)
Professional quality of life Scale Samson et al., 2016 [30]	na	Compassion satisfaction	Very good	0.87 (+)	na	na	Very good	A. 0.68 (−) B. 0.08 (−)
		Secondary traumatic stress		0.82 (+)				
		Burnout		0.69 (−)				
Professional fulfilment Trockel et al., 2018 [31]	na	Work exhaustion	Very good	0.86 (+)	Inadequate	?	Inadequate	?
		Interpersonal disengagement		0.92 (+)				
		Burnout		0.92 (+)				
		Professional fulfilment		0.91 (+)				
Professional fulfilment Fadare et al., 2021 [24]	na	Work exhaustion	Very good	0.92 (+)	na	na	Very good	A. 0.99 (+) B. 0.08 (−)
		Interpersonal disengagement		0.92 (+)				A. 0.99 (+) B. 0.08 (−)
		Professional fulfilment		0.92 (+)				C. 0.07 (+)

Abbreviations: na, not applicable; nr, not reported; Rating measurement properties: (+) sufficient, (−) insufficient, or (?) indeterminate.

## Data Availability

No new data were created or analyzed in this study. Data sharing is not applicable to this article.

## Supplementary Materials

The following supporting information can be downloaded at: https://www.mdpi.com/article/10.3390/ijerph20126114/s1, Supplementary File S1. Search strategy; Supplementary File S2. Risk of bias Quadas-2; Supplementary File S3. Characteristics of the instruments screening for psychological distress.
