# Supplementary material for: Diagnostic Accuracy and Measurement Properties of Instruments Screening for Psychological Distress in Healthcare Workers—A Systematic Review
Source: Int J Environ Res Public Health. 2023 Jun 13;20(12):6114. doi: 10.3390/ijerph20126114 (PMC10298301; doi:10.3390/ijerph20126114)
Supplement: Supplementary file 1 [file ijerph-20-06114-s001.zip › Supplementary file S1. Search strategy.pdf]

Supplementary S1 Search strategy

	**Ovid MEDLINE(R) ALL <1946 to February 09, 2021>**<br>**Search date: 10 February 2021**	
	**Searches**	**Hits**
1	psychometrics/ or psychometr*.mp.	100,169
2	(Physician Well-Being Index or work functioning screener or "WFS-H" or Nurses Work Functioning Questionnaire or Psychiatric Nurse Job Stressor Scale or Nurses? Occupational Stressor Scale or "The Traumatic and Routine Stressors Scale on Emergency Nurses" or "TRSS-EN" or Nursing Stress Scale or Nurse Stress Checklist or Remote Area Nursing Stress Scale or RANSS or Doctors? Job Burnout Questionnaire or Nurse Practitioner Primary Care Organizational Climate Questionnaire or "NP-PCOCQ" or "STRAIN-EOS" or "external observation of work stressors" or Stress of Conscience Questionnaire or Hospital Ethical Climate Scale or Secondary Traumatic Stress Scale).ab,kf,ti. [specific tools]	206
3	(well being index or WBI or burnout battery or "28-item General Health Questionnaire" or "GHQ-28" or Professional Fulfillment Index or pfi scale? or Maslach Burnout Inventory or Oldenburg Burnout Inventory or Maslach Burnout Inventory - Human Services Survey or "MBI-HSS" or Utrecht Work Engagement Scale or "General Health Questionnaire-12" or Brief Job Stress Questionnaire or Job Content Questionnaire or Copenhagen burn out inventory or Spanish Burnout Questionnaire or Granada Burnout Questionnaire).ab,kf,ti. [generic tools]	6216
4	(screen* or detect* or cronbach* or (psychometric* adj15 (valid* or invalid or variat* or invariat* or consist* or inconsist*)) or (analy* adj5 psychometric* propert*) or (valid* and psychometric* propert*) or (psychometric* adj2 valid*)).ab,kf,ti. [early detection]	3,081,893
5	or/1-3	105,949
6	exp employment/ or exp work/ or occupational health/ or occupational groups/ or sick leave/ or exp occupational stress/	183,955
7	(paid work or worker? or occupation* or sick leave or absenteeism or presenteeism or employment or employee? or sickness absence or job? or work abil* or work participa* or work place or work related or work disabil* or work product* or work limit* or work instabili* or work function* or work performance or work capacit or work evaluat* or work direct* or working populat* or workplace or profession*).ab,kf,ti.	800,192
8	6 or 7	895,638
9	(healhcare or health care or hospital* or physician? or nurse? or doctor? or surgeon? or dentist?).ab,hw,kf,ti.	3,113,740
10	(and/4-5,8-9) or 2	2301

	**Ovid Embase Classic+Embase <1947 to 2021 February 09>**<br>**Search date: 10 February 2021**	
	**Searches**	**Hits**
1	psychometry/ or psychometric*.mp.	93,855
2	(Physician Well-Being Index or work functioning screener or "WFS-H" or Nurses Work Functioning Questionnaire or Psychiatric Nurse Job Stressor Scale or Nurses? Occupational Stressor Scale or "The Traumatic and Routine Stressors Scale on Emergency Nurses" or "TRSS-EN" or Nursing Stress Scale or Nurse Stress Checklist or Remote Area Nursing Stress Scale or RANSS or Doctors? Job Burnout Questionnaire or Nurse Practitioner Primary Care Organizational Climate Questionnaire or "NP-PCOCQ" or "STRAIN-EOS" or "external observation of work stressors" or Stress of Conscience Questionnaire or	239

	Hospital Ethical Climate Scale or Secondary Traumatic Stress Scale).ab,kw,ti. [specific tools]	
3	(well being index or WBI or burnout battery or "28-item General Health Questionnaire" or "GHQ-28" or Professional Fulfillment Index or pfi scale? or Maslach Burnout Inventory or Oldenburg Burnout Inventory or Maslach Burnout Inventory - Human Services Survey or "MBI-HSS" or Utrecht Work Engagement Scale or "General Health Questionnaire-12" or Brief Job Stress Questionnaire or Job Content Questionnaire or Copenhagen burn out inventory or Spanish Burnout Questionnaire or Granada Burnout Questionnaire).ab,kw,ti. [generic tools]	8404
4	(screen* or detect* or cronbach* or (psychometric* adj15 (valid* or invalid or variat* or invariat* or consist* or inconsist*)) or (analy* adj5 psychometric* propert*) or (valid* and psychometric* propert*) or (psychometric* adj2 valid*)).ab,kw,ti. [early detection]	4,103,400
5	or/1-3	101,911
6	exp *employment/ or exp *work/ or exp *occupational health/ or exp *healthcare personnel/ or job stress/	756,123
7	(paid work or worker? or occupation* or sick leave or absenteeism or presenteeism or employment or employee? or sickness absence or job? or work abil* or work participa* or work place or work related or work disabil* or work product* or work limit* or work instabili* or work function* or work performance or work capacit or work evaluat* or work direct* or working populat* or workplace or profession*).ab,kw,ti.	1,029,003
8	6 or 7	1,563,539
9	exp healthcare personnel/	1,709,478
10	(healhcare or health care or hospital* or physician? or nurse? or doctor? or surgeon? or dentist?).ab,hw,kw,ti.	5,131,463
11	9 or 10	5,592,338
12	(and/4-5,8,11) or 2	3784
	**Ovid APA PsycInfo <1806 to February Week 1 2021>**<br>**Search date: 10 February 2021**	
	**Searches**	**Hits**
1	psychometrics/ or psychometr*.cw,mp.	145,368
2	(Physician Well-Being Index or work functioning screener or "WFS-H" or Nurses Work Functioning Questionnaire or Psychiatric Nurse Job Stressor Scale or Nurses? Occupational Stressor Scale or "The Traumatic and Routine Stressors Scale on Emergency Nurses" or "TRSS-EN" or Nursing Stress Scale or Nurse Stress Checklist or Remote Area Nursing Stress Scale or RANSS or Doctors? Job Burnout Questionnaire or Nurse Practitioner Primary Care Organizational Climate Questionnaire or "NP-PCOCQ" or "STRAIN-EOS" or "external observation of work stressors" or Stress of Conscience Questionnaire or Hospital Ethical Climate Scale or Secondary Traumatic Stress Scale).ab,id,ti,tm. [specific tools]	329
3	(well being index or WBI or burnout battery or "28-item General Health Questionnaire" or "GHQ-28" or Professional Fulfillment Index or pfi scale? or Maslach Burnout Inventory or Oldenburg Burnout Inventory or Maslach Burnout Inventory - Human Services Survey or "MBI-HSS" or Utrecht Work Engagement Scale or "General Health Questionnaire-12" or Brief Job Stress Questionnaire or Job Content Questionnaire or Copenhagen burn out inventory	4214

	or Spanish Burnout Questionnaire or Granada Burnout Questionnaire).ab,id,ti. [generic tools]	
4	(screen* or detect* or cronbach* or (psychometric* adj15 (valid* or invalid or variat* or invariat* or consist* or inconsist*)) or (analy* adj5 psychometric* propert*) or (valid* and psychometric* propert*) or (psychometric* adj2 valid*)).ab,id,ti,tm. [early detection]	291,383
5	or/1-3	149,279
6	exp occupations/ or exp occupational health/ or occupational exposure/ or occupational health psychology/ or exp working conditions/ or exp occupational stress/	105,108
7	(occupational or working).cw.	40,689
8	(paid work or worker? or occupation* or sick leave or absenteeism or presenteeism or employment or employee? or sickness absence or job? or work abil* or work participa* or work place or work related or work disabil* or work product* or work limit* or work instabili* or work function* or work performance or work capacit or work evaluat* or work direct* or working populat* or workplace or profession*).ab,id,ti.	552,192
9	or/6-8	583,935
10	exp health personnel/	166,430
11	(healhcare or health care or hospital* or physician? or nurse? or doctor? or surgeon? or dentist?).ab,hw,id,ti.	429,167
12	10 or 11	506,975
13	(and/4-5,9,12) or 2	2016
