# Supplementary material for: Diagnostic Accuracy and Measurement Properties of Instruments Screening for Psychological Distress in Healthcare Workers—A Systematic Review
Source: Int J Environ Res Public Health. 2023 Jun 13;20(12):6114. doi: 10.3390/ijerph20126114 (PMC10298301; doi:10.3390/ijerph20126114)
Supplement: Supplementary file 1 [file ijerph-20-06114-s001.zip › Supplementary file S2. Risk of bias Quadas-2.pdf]

Supplementary S2. Risk of bias Quadas-2

**Domain 1 patient selection:**	**Signaling questions**
Could the selection of patients have introduced bias?	1: Was a consecutive or random sample of patients enrolled?<br>2: Was a case-control design avoided?<br>3: Did the study avoid inappropriate exclusions?
Applicability 1 Patient selection:	Are there concerns that the included patients and setting do not match the review question?
**Domain 2 Index test:**	**Signaling questions**
Could the conduct or interpretation of the index test have introduced bias?	1: Were the index test results interpreted without knowledge of the results of the reference standard<br>2: If a threshold was used, was it pre-specified?
Applicability 2 Index test:	Are there concerns that the index test, its conduct, or interpretation differ<br>from the review question?
**Domain 3 Reference standard:**	**Signaling questions**
Could the reference standard, its conduct, or its interpretation have introduced bias?	1: Is the reference standard likely to correctly classify the target condition?<br>2: Were the reference standard results interpreted without knowledge of the results of the index test?
Applicability 3 Reference standard:	Are there concerns that the target condition as defined by the reference<br>standard does not match the question?
**Domain 4 Flow and timing:**	**Signaling questions**
Could the patient flow have introduced bias?	1: Was there an appropriate interval between index test and reference<br>standard?<br>2: Did all patients receive the same reference standard?<br>3: Were all patients included in the analysis?
