# Supplementary material for: Diagnostic Accuracy and Measurement Properties of Instruments Screening for Psychological Distress in Healthcare Workers—A Systematic Review
Source: Int J Environ Res Public Health. 2023 Jun 13;20(12):6114. doi: 10.3390/ijerph20126114 (PMC10298301; doi:10.3390/ijerph20126114)
Supplement: Supplementary file 1 [file ijerph-20-06114-s001.zip › Supplementary file S3. Characteristics of the instruments screening for psychological distress.pdf]

Supplementary file S3. Characteristics of the instruments screening for psychological distress

Instrument	Author, Year	Generic/ specific for HCWs	Target population	Country	Sub-construct	Number of items	Response options	Summary of scores
WFS-H	Boezeman et al., 2016[17]	Specific	Healthcare workers	Netherlan ds	na	6	4-point<br>0 = not at all,<br>1 = slightly agree,<br>2 = agree,<br>3 = totally agree	0−100 Higher score denotes higher impaired work functioning.
Burnout battery	Deng et al., 2017[18]	Specific	Oncology, professionals	China	na	1	7-point symbolic batteries. Full energy to no energy	6 bars means no signs of burnout. Decreasing bars indicate decreasing working energy and the degree of burnout rises.
Physician well-being index	Dyrbye et al., 2013[22]; Dyrbye, et al., 2014[23]; Dyrbye et al., 2019[20]	Specific	Physicians	United states of America	Burnout<br>Depression<br>Stress<br>Fatigue<br>Mental and physical QOL	7	0 = no<br>1 = yes	One point is assigned for each 'yes'. summary scores on the seven-item index range from 0 to 7 (lowest to highest risk for severe distress)
9-item well-being index	Dyrbye et al., 2016[21]	Specific	Physicians	United states of America	Burnout<br>Depression<br>Stress<br>Fatigue<br>Mental and physical QOL	9	0 = no<br>1 = yes	One point is assigned for each "yes"; summary scores on the seven-item index range from 0 to 7 (lowest to highest risk for severe distress).
Professional quality of life Scale	Ang et al., 2020[16]	Generic	Nurses	Singapore	Compassion satisfaction, Burnout,	30	5-point Likert scale<br>1 = never,<br>5 = very often	A higher score in each subscale indicated higher presence of the construct.

					Secondary traumatic stress			
	Galiana et al., 2017 [25]		Professional	Spain	Burnout, Secondary traumatic stress	30	5-point Likert scale 0 = never, 5 = always	nr
			Nurses and palliative Caregiver	Brazil				
	Hemsworth et al., 2018 [28]		Nurses	Canada/ Australia	compassion satisfaction, burnout secondary traumatic stress	30	5-point Likert scale 1 = never, 5 = very often	nr
			Palliative care nurses	Canada				
	Samson et al., 2016[30]		Professionals / physicians, nurses, and social workers	Israel	compassion satisfaction, burnout secondary traumatic stress	30	5-point Likert scale 1 = never, 5 = very often	A higher score in each subscale indicated higher presence of the construct.
Burnout-thriving index	Gates et al., 2019 [26]	Specific	Various HCWs	United states of America	None	1	5 labels: burnout, survival, fine, well, and thriving	Continuum ranging from burnout to thriving, with more neutral conditions in between.
Single burnout item	Hansen et al., 2010 [27]	Generic	Cancer care workers	Australia	None	1	5-point Likert scale 0 = I enjoy my work, 5 = feel completely burned out and often wonder if I can go on. I am at the point where I may need some changes or may	Item dichotomized as no symptoms of burnout and one or more symptoms.
	Dolan et al., 2015 [19]		Registered nurses, licensed practical nurses, medical technicians,	United states of America				

			administrative clerks				need to seek some sort of help	
	Rohland et al., 2004 [29]		Physicians	United states of America				
	Waddimba et al., 2016 [32]		Rural physician/non physician practitioners	United states of America				
Professional fulfillment index	Fadare et al., 2021 [24]	Specific	Pharmacist	United states of America	Professional fulfilment Burnout	16	Subscale professional fulfilment: 0 = not at all true, 4-completely true<br>Subscale work exhaustion and interpersonal disengagement: 0 = not at all true, 4-completely true	Scale scores are calculated by averaging the items scores of all items within each corresponding scale, all scale scores range from 0 to 4.
	Trockel et al., 2018 [31]		Physicians	United states of America	Professional fulfilment Burnout			

Abbreviations: na, not applicable; nr, not reported; WFS-H, work functioning screener; HCWs, healthcare workers; QOL, quality of life
